# Determinants of neonatal mortality among neonates admitted to neonatal intensive care unit of Dessie comprehensive and specialized hospital, Northeast Ethiopia; An unmatched case-control study

**DOI:** 10.3389/fpubh.2022.979402

**Published:** 2022-09-27

**Authors:** Getu Engida Wake, Kalkidan Chernet, Almaz Aklilu, Fentahun Yenealem, Girma Wogie Fitie, Michael Amera Tizazu, Yohannes Moges Mittiku, Moges Sisay Chekole, Geremew Kindie Behulu

**Affiliations:** ^1^Institute of Medicine and Health Science, Department of Midwifery, Debre Berhan University, Debre Birhan, Ethiopia; ^2^College of Medicine and Health Science, Department of Midwifery, Wollo University, Kombolcha, Ethiopia; ^3^College of Medicine and Health Science, Department of Midwifery, Bahir Dar University, Bahir Dar, Ethiopia

**Keywords:** neonatal mortality, determinants, unmatched case-control, Dessie, Ethiopia

## Abstract

**Background:**

According to the World health organization, neonatal mortality is defined as the death of babies within the first 28 days of their lives. The newborn period is the most vulnerable period for a child's survival, with the bulk of neonatal deaths occurring on the first day and week. According to a recent study, about a third of all newborn deaths occur within the first day of life, and nearly three-quarters occur within the first week. This study aimed to assess the determinants of neonatal mortality among neonates admitted to the neonatal intensive care unit in Dessie comprehensive and specialized hospital, northeast Ethiopia.

**Methodology:**

Health institution-based unmatched case-control study was conducted among neonates admitted to Dessie comprehensive and specialized hospital, Ethiopia from February 01 up to March 30, 2020. After keeping cases and controls in separate frames, study participants were chosen using a simple random sampling procedure until the sample size was met. Epi data version 7.0 and SPSS version 25 were used for data entry and analysis respectively. *P* ≤ 0.05 was used as a cut point of statistical significance in multivariable binary logistic regression.

**Results:**

A total of 698 (233 cases and 465 controls) participated in the study. Pregnancy induced hypertension (AOR = 3.02; 95% CI; 1.47–6.17), public hospital delivery (AOR = 3.44; 95% CI; 1.84–6.42), prematurity (AOR = 2.06; 95% CI; 1.43–2.96), being referred (AOR = 4.71; 95% CI; 3.01–7.39), and hypothermia (AOR = 2.44; 95% CI; 1.56–3.82) were determinant factors of neonatal mortality.

**Conclusion:**

Pregnancy-induced hypertension, public hospital delivery, prematurity, referral, and hypothermia were found to be the determinant factors of neonatal mortality. It would be important to give due attention to neonates delivered from mothers with a history of hypertensive disorder. Besides better to give due attention to neonates delivered in public health institutions, prematurely delivered, referred, and hypothermic neonates. Lastly, further research should be conducted to investigate the additional determinants of neonatal mortality.

## Introduction

According to the World Health Organization, neonatal mortality is defined as the death of babies within the first 28 days of their lives (WHO) (1). Very early, early, and late newborn mortality are the three types of neonatal mortality. The deaths that occur on the first day of life and within the first 7 days of life are referred to as very early and early neonatal mortalities. Late neonatal mortality is defined as deaths that occur after the seventh day but before the 28th day of life (1, 2). The newborn period is the most vulnerable period for a child's survival, with the bulk of neonatal deaths occurring on the first day and week, with around one million dying on the first day and over one million dying within the next 6 days (3). The number of neonatal deaths per 1,000 live births in a given year is known as the neonatal mortality rate (NMR), and it is one of the most sensitive indicators of a community's socioeconomic condition, as well as the availability and accessibility of health care services in the country (1, 4).

On a global scale, 2.4 million newborn deaths occurred in the first 28 days, with around 6,700 deaths every day in 2019 (3). According to a recent study, about a third of all newborn deaths occur within the first day of life, and nearly three-quarters occur within the first week (3).

Almost all newborn deaths occurred in developing nations, and the neonatal period is the most dangerous time for a child under the age of five (5). Six of the 12 nations that accounted for two-thirds of all live-born deaths worldwide were in sub-Saharan Africa, and they accounted for around 60% of all neonatal deaths (5, 6).In Ethiopia, over 87,000 newborns die within the first 28 days of life, making it one of the world's highest rates of neonatal mortality (7).

Similarly, neonatal death rates climbed from 29 per 1,000 live births in the Ethiopian demographic health survey (EDHS2016) to 30 per 1,000 live births in the Ethiopian mini demographic health survey (EMDHS 2019) report (8, 9). Goal three of the Sustainable Development Goals (SDG) to end unnecessary death and disability includes reducing newborn mortality (10).

Over the last few decades, considerable progress has been made in reducing newborn death by adopting favorable health policies and allocating adequate resources to accelerate the accomplishment of neonatal survival goals (11–13). In Ethiopia, the integration of reproductive, maternal, newborn, and child health (RMNCH), policy formation, strong leadership, and cooperation, as well as evidence-based interventions, lowered infant death from 49 to 40% Worldwide in 2016 (14). Furthermore, since 2014, a standard neonatal care procedure has been implemented (15) and in Ethiopia, free maternity and neonatal health services were a huge help in lowering neonatal mortality (16). Even though those trials have lowered newborn mortality in several countries (17), Ethiopia's progress was slow, and it fell well short of the UN's ambitious aim of avoiding unnecessary infant deaths and lowering neonatal mortality to 12 per 1,000 live births in every nation by 2030 (8, 9, 18).

The majority of neonatal mortality is caused by preventable and treatable conditions such as diarrhea, pneumonia, sepsis, asphyxia, and preterm, all of which can be avoided by employing basic mother and child health care services (19, 20). Maternal variables such as antepartum hemorrhage, pregnancy-induced hypertension, and other medical/surgical disorders were linked to early infant death, according to another study (21). Low birth weight (LBW), very low birth weight (VLBW), extreme low birth weight (ELBW), and preterm birth are other neonatal and intrapartum concerns to consider (8, 9, 22, 23), prolonged rupture of membrane, mal-presentation (dystocia), home delivery, and instrument delivery (8, 24–26) were positively associated with neonatal mortality. The Amhara region in Ethiopia has the highest infant mortality rate (47/1,000 live births) (8) and the deaths in the NICU were found to be common (23.1%) (27). As a result, determining the determinant variables of newborn mortality is evidence for inventions and a necessary step in reducing the burden of neonatal deaths (4, 28). Despite the seriousness of the situation, there are inadequacies in research on newborn mortality that has been undertaken in Ethiopia, according to our understanding. As a result, we conducted a case-control study to determine the determinants of neonatal death among neonates hospitalized in the neonatal critical care unit at Dessie Comprehensive Specialized Hospital in Northeast Ethiopia.

## Methods

### Study area and period

From February 01 up to March 30, 2020, the research was carried out at Dessie Comprehensive and Specialized Hospital. Dessie is the capital city of the South Wollo Zone and is located 401 kilometers from Ethiopia's capital, Addis Ababa. The hospital is one of Ethiopia's Comprehensive Specialized Level Hospitals, serving more than 8 million people in Northeast Ethiopia. Since September 2012, it has provided neonatal intensive care unit (NICU) services and currently, the facility is divided into three sections: Kangaroo Mother Care (KMC), term, and preterm. The facility has 37 beds, five phototherapy machines, five incubators, 12 radiant warmers, four heaters, three mechanical ventilators, three oxygen concentrators, and 11 oxygen cylinders filled every 15 days. Continuous positive airway pressure (CPAP) was also made with locally available items such as a Ringer's lactated solution bag, tap water, and an oxygen cylinder.

### Population

#### Source population

All neonates, admitted to the NICU of Dessie Comprehensive and Specialized Hospital, were a source population.

#### Study population

##### Case

All neonates, those were admitted to the NICU of Dessie comprehensive and specialized hospital and had a death summary from February 01 up to March 30, 2020.

##### Control

All neonates admitted to NICU of Dessie comprehensive and specialized hospital, who were discharged alive from February 01 up to March 30, 2020.

### Sample size determination and sampling method

#### Sample size determination

The sample size for an unmatched case-control study was estimated using Epi data software version 7.0 under the assumption of a double population proportion formula, taking into account the following parameters: The maximum sample size was obtained by using low birth weight (LBW) as a determinant factor from a previous study conducted in Adama, Ethiopia, where the proportion of exposure among cases was 60% and among controls was 36% with an odds ratio of 1.6 and different sample sizes were produced from previously identified determinants of neonatal mortality (19). Based on the provided data, the maximum final sample size was 698 (233 cases and 465 controls).

#### Sampling technique

**Figure 1:**
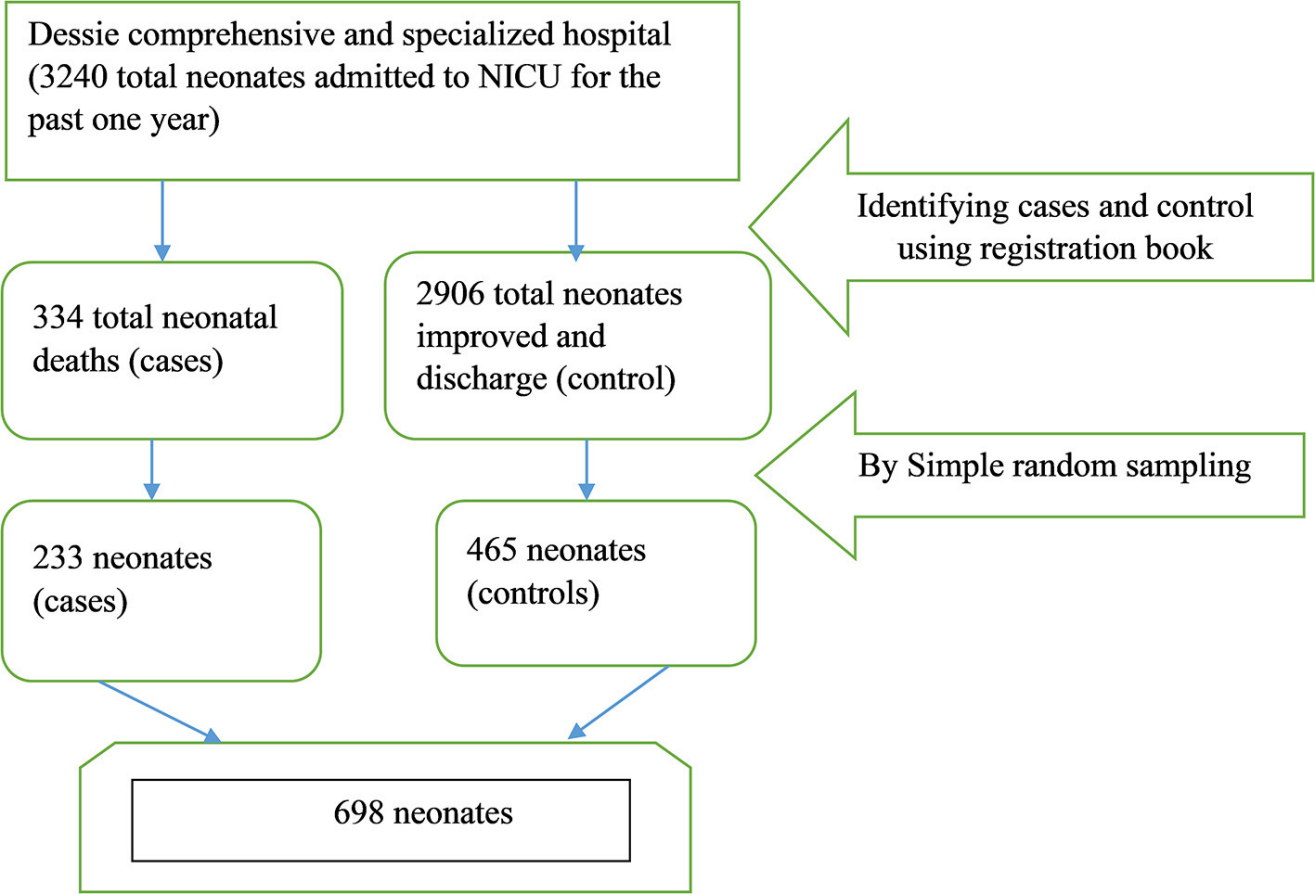
A diagram of the sampling technique among NICU admitted newborns in Dessie Comprehensive and Specialized Hospital was presented *from February 1/2020 to March 30/2020*.

A registration book from a neonatal intensive care unit (NICU) was used to select study participants. Then, after keeping cases and controls in separate frames, the final research participants were chosen using a simple random sampling procedure from a list of case and control groups received from the registration book until the sample size was met. Two controls were randomly selected from the frame of the register for each case using a computer-generated random sampling approach, and this procedure was repeated until the needed sample size was reached Figure 1.

### Study variable

#### Dependent variable

Neonatal mortality.

#### Independent variable

**Maternal characteristics:** Age, parity, antenatal care visit, pregnancy and delivery complications such as pregnancy-induced hypertension, antepartum hemorrhage, preterm rupture membrane, and site and mode of delivery are all factors to consider.

Neonatal characteristics include gestational age at birth, weight, birth type, breastfeeding within 1 h of birth, referred neonate, referral location, the temperature at admission time, and cause for NICU hospitalization [congenital anomaly, asphyxia, sepsis, jaundice, respiratory distress syndrome (RDS), meconium aspiration syndrome (MAS)].

### Data collection procedure

Data were collected through chart reviews using a pre-tested checklist adapted from kinds of literature (29–32). The checklist includes three sections; maternal-related characteristics, and neonatal-related variables. The data were collected by a five-degree pharmacy using an English version checklist under strict daily supervision. The supervision was done by the Principal investigator and one M.Sc. Midwife Supervisor.

### Data quality control

Before being utilized to collect the real data, the checklist was converted to English language and pretested on 35 neonates at Dessie comprehensive and specialized hospital. Data collectors and supervisors received a 1-day training session on the data collection tool and procedure. During data collection, the questionnaires were given codes. The principal investigator and supervisor kept a close eye on the data to ensure that it was complete and consistent during the data collection period.

### Data analysis

Data were cleaned, coded, and put into Epi data version 7.0 before being exported to SPSS version 25. For the cases and controls, summary statistics such as median and Interquartile Range (IQR) were calculated. The independent variables were compared between cases and controls in a cross-tabulation. Then, to analyze the crude relationship between the independent and outcome variables, bivariable binary logistic regression was used. To analyze the net effect by controlling confounders, factors having a *P* < 0.25 in bivariable binary logistic regression were put into a multivariable binary logistic regression. In multivariable binary logistic regression, factors with a *p* < 0.05 were considered statistically significant determinants of Neonatal Mortality.

## Results

### Maternal and neonatal characteristics

A total of 233 cases and 465 controls were included in this investigation. Approximately (84.1%) of cases and (85.6%) of controls were delivered by women aged 20–34. The majority of mothers in both cases (98.3%) and controls (97.2%) had an antenatal care (ANC) visit. Premature rupture of membrane occurred in about (11.6%) of cases and (7.5%) of controls. More than half of the cases (53.6%) and controls (57.8%) were delivered naturally through the vaginal canal. Preterm newborns made up around half of the cases (51%) and roughly (27.5%) of the controls. Low birth weight was seen in more than half of the cases (54.9%) and 151 (32.5%) of the controls. The majority of cases (85.4%) and controls (82.6%) were singleton births. Hypothermia affected more than half of the cases (77.3%) and 270 (58.1%) of the controls. About (16.7%) of cases and (13.3%) of controls had asphyxia. Respiratory distress syndromes (RDS) were found in less than half of the cases (33.5%) and 103 (22.2%) of the controls (Table 1).

**Table 1:** Maternal and neonatal related factors for neonates admitted to neonatal intensive care unit of Dessie comprehensive and specialized hospital, Northeast Ethiopia, 2020 (*n* = 698).

**Variables**	**Neonatal mortality**	
	**Cases** **(*n* = 233)**	**Controls** **(*n* = 465)**
	***N*** **(%)**	***N*** **(%)**
**Age of the mother (in years)**		
<20	11 (4.7)	18 (3.9)
20–34	196 (84.1)	398 (85.6)
>34	26 (11.2)	49 (10.5)
**Parity**		
Prim Para	131 (56.2)	270 (58.1)
Multi Para	89 (38.2)	175 (37.6)
Grand multi Para	13 (5.6)	20 (4.3)
**ANC Visit**		
Yes	229 (98.3)	452 (97.2)
No	4 (1.7)	13 (2.0)
**PROM**		
Yes	27 (11.6)	35 (7.5)
No	206 (88.4)	430 (92.5)
**Antepartum Hemorrhage**		
Yes	45 (19.3)	96 (20.6)
No	188 (80.7)	369 (79.4)
**Hypertensive disorder**		
Yes	22 (9.4)	15 (3.4)
No	211 (90.6)	449 (96.6)
**Place of delivery**		
Home	6 (2.6)	6 (1.3)
Health Center	46 (19.7)	107 (23.0)
Public Hospital	157 (67.4)	299 (64.3)
Private	24 (10.3)	53 (11.4)
**Mode of delivery**		
SVD	125 (53.6)	269 (57.8)
C/S	72 (30.9)	119 (25.6)
Instrumental	36 (15.5)	77 (16.6)
**GA/weeks**		
< 37	119 (51.1)	128 (27.5)
≥37	114 (48.9)	337 (72.5)
**Birth weight**		
< 2,500	128 (54.9)	151 (32.5)
≥2,500	105 (45.1)	314 (67.5)
**Type of birth**		
Single	199 (85.4)	384 (82.6)
Multiple	34 (14.6)	81 (17.4)
**Referred neonate**		
Yes	160 (68.7)	228 (49.0)
No	73 (31.3)	237 (51.0)
**Place of referral (*****n*** **= 388)**		
Public Hospital	85 (53.1)	63 (27.6)
Health center	52 (32.5)	112 (49.1)
Private	23 (14.4)	53 (23.2)
**Breastfeed within 1 h**		
Yes	71 (30.5)	168 (36.1)
No	162 (69.5)	297 (63.9)
**Temperature/in degrees Celsius**		
< 36.5	180 (77.3)	270 (58.1)
36.5–37.5	38 (16.3)	155 (33.3)
>37.5	15 (6.4)	40 (8.6)
**Asphyxia**		
Yes	39 (16.7)	62 (13.3)
No	194 (83.3)	403 (86.7)
**Jaundice**		
Yes	7 (3.0)	14 (3.0)
No	226 (97.0)	451 (97.0)
**Congenital anomaly**		
Yes	5 (2.1)	11 (2.4)
No	228 (97.9)	454 (97.6)
**Sepsis**		
Yes	28 (12.0)	58 (12.5)
No	205 (88.0)	407 (87.5)
**Respiratory distress**		
Yes	78 (33.5)	103 (22.2)
No	155 (66.5)	362 (77.8)
**MAS**		
Yes	50 (21.5)	87 (18.7)
No	183 (78.5)	378 (81.3)

### Determinants of neonatal mortality

In bivariable binary logistic regression analyses variables such as neonates delivered from mothers who had a history of the premature rupture of membrane and pregnancy-induced hypertension, place of delivery, mode of delivery, gestational age, birth weight, referred neonates, Breastfeeding initiation time, presence of hypothermia, asphyxia and respiratory distress syndrome (RDS) were significantly associated with neonatal mortality. After controlling for potential confounders on multivariable binary logistic regression analysis; neonates delivered from mothers who had pregnancy-induced hypertension, Place of delivery, prematurity, referred neonates, and presence of hypothermia were identified as determinant factors of neonatal mortality. Accordingly, neonates delivered from mothers who had pregnancy-induced hypertension were almost 3 times more likely to face neonatal mortality compared to neonates who were delivered from mothers who had no pregnancy-induced hypertension (AOR = 3.02; 95% CI; 1.47–6.17). Neonates delivered at the public hospital were almost 3 times higher odds of neonatal mortality as compared to neonates who were delivered at private hospitals (AOR = 3.44; 95% CI; 1.84–6.42). Preterm babies were 2 times more prone to neonatal mortality than those term babies (AOR = 2.06; 95% CI; 1.43–2.96). Referred neonates were almost 5 times at higher odds of neonatal mortality as compared to neonates who were not referred (AOR = 4.71; 95% CI; 3.01–7.39). The odds of neonatal mortality among hypothermic newborns were 2 times higher as compared to non-hypothermic newborns (AOR = 2.44; 95% CI; 1.56–3.82) (Table 2).

**Table 2:** Determinants of neonatal mortality among neonates admitted to neonatal intensive care unit of Dessie comprehensive and specialized hospital, northeast Ethiopia, 2020 (*n* = 698).

**Variables**	**Neonatal mortality**		**COR (95%CI)**	**AOR (95% CI)**
	**Cases (*n* = 233)**	**Controls (*n* = 465)**		
	***N*** **(%)**	***N*** **(%)**		
**PROM**				
Yes	27 (11.6)	35 (7.5)	1.61 (0.95–2.73)	
No	206 (88.4)	430 (92.5)	1	
**Hypertensive disorder**				
Yes	22 (9.4)	16 (3.4)	2.92 (1.50–5.68)	3.02 (1.47–6.17)^⋆^
No	211 (90.6)	449 (96.6)	1	1
**Place of delivery**				
Home	6 (2.6)	6 (1.3)	2.20 (0.64–7.55)	1.97 (0.54–7.18)
Health center	46 (19.7)	107 (23.0)	0.94 (0.52–1.72)	1.12 (0.60–2.11)
Public hospital	157 (67.4)	299 (64.3)	1.16 (0.69–1.95)	3.44 (1.84–6.42)^⋆⋆^
Private	24 (10.3)	53 (11.4)	1	1
**Mode of delivery**				
SVD	125 (53.6)	269 (57.8)	1	
C/S	72 (30.9)	119 (25.6)	1.30 (0.91–1.87)	
Instrumental delivery	77 (16.6)	36 (15.5)	1.01 (0.64–1.58)	
**GA**				
< 37	119 (51.1)	128 (27.5)	2.75 (1.98–3.81)	2.06 (1.43–2.96)^⋆⋆^
≥37	114 (48.9)	337 (72.5)	1	1
**Birth weight**				
< 2,500	128 (54.9)	151 (32.5)	2.53 (1.83–3.50)	
≥2,500	105 (45.1)	314 (67.5)	1	
**Referred neonate**				
Yes	160 (68.7)	228 (49.0)	2.28 (1.63–3.17)	4.71 (3.01–7.39)^⋆⋆^
No	73 (31.3)	237 (51.0)	1	1
**Breastfed within 1 h**				
Yes	71 (30.5)	168 (36.1)	1	
No	162 (69.5)	297 (63.9)	1.29 (0.92–1.81)	
**Temperature at admission**				
< 36.5	180 (77.3)	270 (58.1)	2.72 (1.82–4.06)	2.44 (1.56–3.82)^⋆⋆^
36.5–37.5	38 (16.3)	155 (33.3)	1	1
>37.5	15 (6.4)	40 (8.6)	1.53 (0.76–3.05)	1.04 (0.49–2.19)
**Asphyxia**				
Yes	39 (16.7)	62 (13.3)	1.31 (0.84–2.02)	
No	194 (83.3)	403 (86.7)	1	
RDS				
Yes	78 (33.5)	103 (22.2)	1.77 (1.25–2.51)	
No	155 (66.5)	362 (77.8)	1	

⋆ p < 0.05;

⋆⋆ p < 0.001; 1,

Reference; AOR, Adjusted Odd Ratio, CI, Confidence Interval; COR, crude odds ratio.

## Discussions

Although the reason for neonatal mortality is unknown, it is a multifaceted issue. This study looked at some of the key factors that influence neonatal mortality in neonates admitted to Dessie's comprehensive and specialized hospital's NICU. According to the findings, neonates born to women with pregnancy-induced hypertension, place of delivery, preterm, referral, and hypothermia were all factors that contributed to neonatal mortality. The odds of neonatal death were three times more likely among neonates delivered from mothers who had pregnancy-induced hypertension in comparison to their counterparts. This finding was consistent with the results of a study conducted in the Netherlands and two studies in Ethiopia (33–35). Maternal hypertensive disorders during the pregnancy increase the risk of low birth weight, low 1st minute Apgar score, respiratory distress syndrome, and preterm birth which were the leading cause of neonatal mortality (33). Another piece of evidence indicated that small size neonates were highly susceptible to different infections due to having low immunity defense (33).

Neonates delivered at public health institutions had three times higher odds of neonatal mortality as compared to neonates who were delivered at private health institutions. This result was in line with the results of the study conducted in Australia (36). This might be because of the high coverage of birth in public health institutions as compared to private health institutions. On and another hand delay in the decision to seek care, delay in reaching care, and delay in receiving adequate health care for new babies by the doctor in governmental health institutions since they are engaged with various private institutions for pecuniary benefits and hand over the duty to new untrained doctors and other health care professionals. The odds of neonatal death were two times higher among preterm-delivered neonates as compared to term-delivered neonates. This finding is consistent with a study conducted in Brazil, Kenya, and four studies in Ethiopia (24, 31, 32, 34, 37–39). Prematurity is related to difficulty in extra uterine adaptation due to the immaturity of different organ systems (39). The supply of oxygen through the placenta increases with the course of gestation according to the fetal needs (40, 41). Premature birth interrupts this process, exposing the newborn to oxygen deprivation, which can be harmful to the tissues, especially the nervous system, increasing the risk of cerebral palsy, visual disturbances, and chronic disease in adulthood (42). Another piece of evidence indicated that preterm and low birth weight babies were more likely to be prone to complications such as hypothermia, infections, and birth asphyxia (resulting in tissue hypoxia and multi-organ failure) (31).

The odds of neonatal mortality among referred neonates were almost five times higher compared to neonates who were not referred. This finding was similar to the results of a study done in India, a low resource setting, and Ethiopia (43–45). This might be due to delays in deciding to seek care, delay in reaching a first referral level facility, and delay in actually receiving care after arriving at the facility. Another piece of evidence indicated that lack of well-equipped infrastructure to respond to a crisis, non-availability of obstetric emergency care, and lack of transportation facilities to deal with emergency cases add to the loss of lives (46). The odds of neonatal death among hypothermia neonates were almost five times higher compared to neonates who had normal body temperature. This finding was similar to the results of a study conducted in India, Ethiopia south Nepal (43, 47, 48). This can be justified by neonates who are in a hypothermic state may be more prone to different infections and are more likely to become septic and die when compared to neonates with normal body temperatures (49).

## Limitations

Our study didn't include other maternal socio-demographic characteristics such as the number of prenatal care received, the history of preterm birth before the delivery of the current newborn, and type of birth attendant (Physician, Midwife, and Nurse) and social determinants except for maternal age. Besides, professional-related factors were not incorporated in this study due to the COVID-19 endemic.

## Conclusion and recommendation

Neonates delivered from mothers who had a history of pregnancy-induced hypertension, neonates delivered in Public hospitals, being prematurity, being referred and Hypothermia was found to be the determinant factors of neonatal mortality among NICU admitted neonates. It would be important to give due attention to neonates delivered from mothers with a history of hypertensive disorder. Besides better to give due attention to neonates delivered in public health institutions, prematurely delivered, referred, and hypothermic neonates. Lastly, further research should be conducted to investigate the additional determinants of neonatal mortality.

## Data availability statement

The original contributions presented in the study are included in the article/supplementary material, further inquiries can be directed to the corresponding authors.

## Ethics statement

Helsinki's declaration for medical research involving human subjects was followed. Ethical clearance was obtained from the institutional health research review committee board (Ref. No. IRB-014/02/2020) of the College of Medicine and Health Sciences of Bahir Dar University. Permission was obtained from all responsible bodies at Dessie Comprehensive and Specialized Hospital, NICU, and client registration (card room).

## Author contributions

GEW, KC, AA, FY, GW, YM, MA, MS, and GB: conceptualization. GEW, GW, KC, FY, and GB: methodology. GW, AA, FY, YM, and MS: software and supervision. GEW, MA, KC, GW, and GB: formal analysis. GW, MA, AA, YM, MS, and GB: data curation. GEW, GW, FY, and GB: writing—original draft preparation. GEW, GW, KC, and MA: writing—review and editing. GEW, AA, and FY: visualization. GEW, GW, YM, MA, MS, and GB: funding acquisition. All authors have read and approved the final version of the manuscript to be published.

## Funding

Bahir Dar University had covered the costs for data collection instruments, data collectors, and supervisors, but the funder had no role in the decision to publish.

## Conflict of interest

The authors declare that the research was conducted in the absence of any commercial or financial relationships that could be construed as a potential conflict of interest.

## Publisher's note

All claims expressed in this article are solely those of the authors and do not necessarily represent those of their affiliated organizations, or those of the publisher, the editors and the reviewers. Any product that may be evaluated in this article, or claim that may be made by its manufacturer, is not guaranteed or endorsed by the publisher.

## Abbreviations

ANC: Antenatal Care
AOR: Adjusted odds ratio
CI: Confidence interval
EDHS: Ethiopian Demographic and Health Survey
LBW: Low birth weight
MAS: Meconium aspiration syndrome
NICU: Neonatal intensive care unit
NM: Neonatal mortality
NMR: Neonatal mortality rate
OR: Odds ratio
RDS: Respiratory distress syndrome
SDG: Sustainable development goal
SPSS: Statistical package for social science
WHO: World health organization.

## References

[1] World health Organization. Neonatal and Perinatal Mortality,Country, Regional and Global Estimates. (2006). p. 433–43.

[2] Barfield WD. Standard terminology for fetal, infant, and perinatal deaths. Pediatrics. (2016) 137:e20160551. 10.1542/peds.2016-0551 27244834

[3] UNICEF, WHO, WBO. Levels & Trends in Child Mortality Report. (2020). Un Igme. (2020). p. 48. Available online at: https://www.unicef.org/media/79371/file/UN-IGME-child-mortality-report-2020.pdf.pdf

[4] World Health Organisation (WHO). Making Every Baby Count: Audit and Review of Stillbirths and Neonatal Deaths. WHO Libr Cat Data (2016). p. 144.

[5] UN IGME. Levels & Trends in Child Mortality: Estimates: Report (2018). Who/Unicef/World Bank/Un. (2018). 1–48.

[6] World Health Organization. Every newborn: An action plan to end Preventable deaths. Who, Unicef. (2014). 58. Available online at: https://www.who.int/initiatives/every-newborn-action-plan

[7] Region P. Maternal and Newborn Health in Southern. (2020). (February 2018):1–166.

[8] Demographich and health survey ethiopia 2016. (2016). 115–123 p.

[9] Ethiopian Public Health Institute (EPHI) ICF. Ethiopia Mini Demographic and Health Survey 2019: Final Report. (2019). Available online at: https://dhsprogram.com/pubs/pdf/FR363/FR363. p. 1–207

[10] Bora JK Saikia N. Neonatal and under-five mortality rate in Indian districts with reference to Sustainable Development Goal 3: an analysis of the National Family Health Survey of India (NFHS), 2015–2016. PLoS ONE. (2018) 13:e0201125. 10.1371/journal.pone.0201125 30059555 PMC6066210

[11] World Health Statistics. World Heal Organization. (2019). p. 1–19.

[12] WHO UNICEF. Reaching the Every Newborn National. (2020). Milestones Country Progress, Plans and Moving Forward (2017). p. 2015–2018. Available online at: http://apps.who.int/iris/bitstream/10665/255719/1/9789241512619-eng.pdf?ua=1

[13] IEG World Bank ICF MIGA. Delivering the Millennium Development Goals to Reduce Maternal and Child Mortality: A Sytematic Review of Impact Evaluation Evidence. (2012). p. 149. Available online at: https://www.oecd.org/derec/norway/WORLDBANKDeliveringtheMDGtoreducematernalandchildmortality.pdf

[14] UNICEF, WHO, The World Bank Group UN Population Division. Levels and Trends in Child Mortality: Report. (2017). United Nations Inter-gr Child Mortal Estim (2017). p. 40. Available online at: https://childmortality.org/wp-content/uploads/2018/10/UN-IGME-Child-Mortality-Report-2017.pdf

[15] Sava DI. Neonatal intensi (NICU) Training Management Protocolve care unit. OT Pract. (2014) 13:8. Available online at: http://repository.iifphc.org/bitstream/handle/123456789/709/NICU%20Training%20Management%20Protocol_08April%202014%20.pdf?sequence=1&isAllowed=y

[16] Ministry of Health Ethiopia, PMNCH, World Health Organization, World Bank AHPSR. Success Factors for Women's and Children's Health Ethiopia. (2015). p. 28.

[17] World Health Organization (WHO). World Health Statics. Vol. 3, Khatulistiwa Informatika. (2015). p. 124–133.

[18] United Nations. Sustainable Development Goal 2015–2030. (2020).

[19] Jehan I Harris H Salat S Zeb A Mobeen N Pasha O. Neonatal mortality, risk factors and causes: a prospective population-based cohort study in urban Pakistan. Bull World Health Organ. (2009) 87:130–8. 10.2471/BLT.08.050963 19274365 PMC2636189

[20] Debelew GT Afework MF Yalew AW. Determinants and causes of neonatal mortality in jimma Zone, Southwest Ethiopia: a multilevel analysis of prospective follow up study. PLoS ONE. (2014) 9:e107184. 10.1371/journal.pone.0107184 25232842 PMC4169420

[21] Vogel JP Souza JP Mori R Morisaki N Lumbiganon P Laopaiboon M. Maternal complications and perinatal mortality. Obstet Anesth Dig. (2015) 35:73. 10.1097/01.aoa.0000463817.02019.7d 24641538

[22] Abdallah Y Namiiro F Mugalu J Nankunda J Vaucher Y McMillan D. Is facility based neonatal care in low resource setting keeping pace? A glance at Uganda's National Referral Hospital. Afr Health Sci. (2016) 16:347–55. 10.4314/ahs.v16i2.2 27605949 PMC4994572

[23] Reyesa JCL Ramírez ROP Ramosa LL Ruiz LMG Vázquez EAB Patinõ VR. Neonatal mortality and associated factors in newborn infants admitted to a Neonatal Care Unit. Arch Argent Pediatr. (2018) 116:42–8. 10.5546/aap.2018.eng.42 29333811

[24] Yego F D'Este C Byles J Nyongesa P Williams JS. A case-control study of risk factors for fetal and early neonatal deaths in a tertiary hospital in Kenya. BMC Pregnancy Childbirth. (2014) 14:389. 10.1186/s12884-014-0389-8 25432735 PMC4298961

[25] Abdullah A Hort K Butu Y Simpson L. Risk factors associated with neonatal deaths: a matched case-control study in Indonesia. Glob Health Action. (2016) 9:30445. 10.3402/gha.v9.30445 26895147 PMC4759830

[26] Demisse AG Alemu F Gizaw MA Tigabu Z. Patterns of admission and factors associated with neonatal mortality among neonates admitted to the neonatal intensive care unit of University of Gondar Hospital, Northwest Ethiopia. Pediatr Health Med Ther. (2017) 8:57–64. 10.2147/PHMT.S130309 29388628 PMC5774602

[27] Mehretie K. Institution Based Prospective Cross-Sectional Study on Patterns of Neonatal Morbidity at Gondar University Hospital Neonatal Unit. (2011). p. 73–9. 26949319 10.4314/ejhs.v26i1.12 PMC4762962

[28] Mengesha HG Sahle BW. Cause of neonatal deaths in Northern Ethiopia: a prospective cohort study. BMC Public Health. (2017) 17:1–8. 10.1186/s12889-016-3979-8 28077109 PMC5225539

[29] Kolobo H Chaka T Kassa R. Determinants of neonatal mortality among newborns admitted to neonatal intensive care unit Adama, Ethiopia: A case–control study. J Clin Neonatol. (2019) 8:232. 10.4103/jcn.JCN_23_19

[30] Alebel A Wagnew F Petrucka P Tesema C Moges NA Ketema DB. Neonatal mortality in the neonatal intensive care unit of Debre Markos referral hospital, Northwest Ethiopia: A prospective cohort study. BMC Pediatr. (2020) 20:1–11. 10.1186/s12887-020-1963-z 32061260 PMC7023807

[31] Desalew A Sintayehu Y Teferi N Amare F Geda B Worku T. Cause and predictors of neonatal mortality among neonates admitted to neonatal intensive care units of public hospitals in eastern Ethiopia: a facility-based prospective follow-up study. BMC Pediatr. (2020) 20:1–11. 10.1186/s12887-020-02051-7 32290819 PMC7155275

[32] Seid SS Ibro SA Ahmed AA Olani Akuma A Reta EY Haso TK. Causes and factors associated with neonatal mortality in Neonatal Intensive Care Unit (NICU) of Jimma University Medical Center, Jimma, South West Ethiopia. Pediatr Heal Med Ther. (2019) 10:39–48. 10.2147/PHMT.S197280 31191085 PMC6519704

[33] van Esch JJA van Heijst AF de Haan AFJ van der Heijden OWH. Early-onset preeclampsia is associated with perinatal mortality and severe neonatal morbidity. J Matern Neonatal Med. (2017) 30:2789–94. Available from: 10.1080/14767058.2016.1263295 28282780

[34] Kidus F Woldemichael K Hiko D. Predictors of neonatal mortality in Assosa zone, Western Ethiopia. BMC Pregnancy Childbirth. (2019) 19:1–13. 10.1186/s12884-019-2243-5 30925903 PMC6441179

[35] Berhan Y Endeshaw G. Maternal mortality predictors in women with hypertensive disorders of pregnancy: a retrospective cohort study. Ethiop J Health Sci. (2015) 25:89–98. 10.4314/ejhs.v25i1.12 25733789 PMC4337086

[36] Jang W Flatley C Greer RM Kumar S. Comparison between public and private sectors of care and disparities in adverse neonatal outcomes following emergency intrapartum cesarean at term – a retrospective cohort study. PLoS ONE. (2017) 12:187040. 10.1371/journal.pone.0187040 29149182 PMC5693444

[37] De Souza S Duim E Nampo FK. Determinants of neonatal mortality in the largest international border of Brazil: a case-control study. BMC Public Health. (2019) 19:1–9. 10.1186/s12889-019-7638-8 31619198 PMC6796356

[38] Roro EM Sisay MM Sibley LM. Determinants of perinatal mortality among cohorts of pregnant women in three districts of North Showa zone, Oromia Region, Ethiopia: Community based nested case control study. BMC Public Health. (2018) 18:1–11. 10.1186/s12889-018-5757-2 30021557 PMC6052561

[39] FMOH. Neonatal Intensive Care Unit (NICU) Training Participants' Manual. (2014). p. 194.

[40] de Oliveira TG Freire PV Moreira FT de Moraes J da SB Arrelaro RC Ricardi SRVA. Apgar score and neonatal mortality in a hospital located in the southern area of São Paulo City, Brazil. Einstein. (2012) 10:22–8. 10.1590/S1679-45082012000100006 23045821

[41] Iliodromiti S MacKay DF Smith GCS Pell JP Nelson SM. Apgar score and the risk of cause-specific infant mortality: a population-based cohort study. Lancet. (2014) 384:1749–55. 10.1016/S0140-6736(14)61135-1 25236409

[42] Mwaniki MK Atieno M Lawn JE Newton CRJC. Long-term neurodevelopmental outcomes after intrauterine and neonatal insults: a systematic review. Lancet. (2012) 379:445–52. 10.1016/S0140-6736(11)61577-8 22244654 PMC3273721

[43] Aggarwal K Gupta R Sharma S Sehgal R Roy M. Mortality in newborns referred to tertiary hospital: an introspection. J Fam Med Prim Care. (2015) 4:435. 10.4103/2249-4863.161348 26288788 PMC4535110

[44] Cavallin F Bonasia T Yimer DA Manenti F Putoto G Trevisanuto D. Risk factors for mortality among neonates admitted to a special care unit in a low-resource setting. BMC Pregnancy Childbirth. (2020) 20:1–8. 10.1186/s12884-020-03429-2 33228644 PMC7686767

[45] Mihiretu A Negash T Elazar T. Perinatal death and associated factors in wolaita sodo referral hospital, Southern Ethiopia: a Facility based cross-sectional study. Prim Heal Care Open Access. (2017) 7, 1–5. 10.4172/2167-1079.1000269

[46] Arokiasamy P Gautam A. Neonatal mortality in the empowered action group states of India: trends and determinants. J Biosoc Sci. (2008) 40:183–201. 10.1017/S0021932007002623 18093346

[47] Orsido TT Asseffa NA Berheto TM. Predictors of Neonatal mortality in Neonatal intensive care unit at referral Hospital in Southern Ethiopia: A retrospective cohort study. BMC Pregnancy Childbirth. (2019) 19:1–9. 10.1186/s12884-019-2227-5 30819143 PMC6396455

[48] Mullany LC Katz J Khatry SK LeClerq SC Darmstadt GL Tielsch JM. Neonatal hypothermia and associated risk factors among newborns of southern Nepal. BMC Med. (2010) 8:43. 10.1186/1741-7015-8-43 20615216 PMC2911389

[49] Woday Tadesse A Mekuria Negussie Y Aychiluhm SB. Neonatal mortality and its associated factors among neonates admitted at public hospitals, pastoral region, Ethiopia: A health facility based study. PLoS ONE. (2021) 16:e0242481. 10.1371/journal.pone.0242481 33730039 PMC7968682

